# The First Non-LRV RNA Virus in *Leishmania*

**DOI:** 10.3390/v12020168

**Published:** 2020-02-02

**Authors:** Danyil Grybchuk, Diego H. Macedo, Yulia Kleschenko, Natalya Kraeva, Alexander N. Lukashev, Paul A. Bates, Pavel Kulich, Tereza Leštinová, Petr Volf, Alexei Y. Kostygov, Vyacheslav Yurchenko

**Affiliations:** 1 Life Science Research Centre, Faculty of Science, University of Ostrava, 71000 Ostrava, Czech Republic; danilaman@gmail.com (D.G.); diegohqm@gmail.com (D.H.M.); luzikhina@gmail.com (N.K.); 2 Central European Institute of Technology, Masaryk University, 60177 Brno, Czech Republic; 3 Martsinovsky Institute of Medical Parasitology, Sechenov University, Moscow 119435, Russia, alexander_lukashev@hotmail.com (A.N.L.); 4 Division of Biomedical and Life Sciences, Faculty of Health and Medicine, Lancaster University, Lancaster LA1 4YE, UK; p.bates@lancaster.ac.uk; 5 Laboratory of Electron Microscopy, Veterinary Research Institute, 62100 Brno, Czech Republic; kulich@vri.cz; 6 Department of Parasitology, Faculty of Science, Charles University, 12844 Prague, Czech Republic; Terka.Kratochvilova@seznam.cz (T.L.); volf@cesnet.cz (P.V.); 7 Laboratory of Cellular and Molecular Protistology, Zoological Institute of the Russian Academy of Sciences, St. Petersburg 199034, Russia

**Keywords:** Bunyavirales, Leishmania martiniquensis, leishbunyavirus

## Abstract

In this work, we describe the first *Leishmania*-infecting leishbunyavirus—the first virus other than *Leishmania RNA virus* (LRV) found in trypanosomatid parasites. Its host is *Leishmania martiniquensis*, a human pathogen causing infections with a wide range of manifestations from asymptomatic to severe visceral disease. This virus (*Lmar*LBV1) possesses many characteristic features of leishbunyaviruses, such as tripartite organization of its RNA genome, with ORFs encoding RNA-dependent RNA polymerase, surface glycoprotein, and nucleoprotein on L, M, and S segments, respectively. Our phylogenetic analyses suggest that *Lmar*LBV1 originated from leishbunyaviruses of monoxenous trypanosomatids and, probably, is a result of genomic re-assortment. The *Lmar*LBV1 facilitates parasites’ infectivity in vitro in primary murine macrophages model. The discovery of a virus in *L. martiniquensis* poses the question of whether it influences pathogenicity of this parasite in vivo, similarly to the LRV in other *Leishmania* species.

## 1. Introduction

*Bunyavirales* is an order of negative-sense single-stranded RNA (-ssRNA) viruses [1]. They typically have three genomic segments (large, L; medium, M; small, S) encoding a viral RNA-dependent RNA polymerase L (RDRP L), a surface glycoprotein precursor, and a nucleoprotein, respectively [2]. Additional ORFs, usually involved in counteracting the host antiviral response, may be present in S or M segments [3,4]. Each viral segment has terminal complementary sequences governing its interaction with the polymerase. Furthermore, multiple molecules of a nucleoprotein wrap around genomic RNA following helical symmetry [4]. Together, an RNA molecule, a polymerase, and the nucleoproteins form a functional viral ribonucleoprotein (vRNP) capable of transcription and replication [5]. Virions are usually 90–100 nm in diameter and consist of vRNPs of each genomic segment enclosed by a lipid membrane with incorporated viral glycoproteins [3]. Many bunyaviruses (a generic term for *Bunyavirales*) are causative agents of arthropod-borne diseases of vertebrates and plants [6].

Recent metatranscriptomic studies revealed a plethora of deep branching bunyaviruses from vertebrates and invertebrates, suggesting a long-term coevolution of these viruses with their hosts and vectors [7,8,9]. Of note, some bunyaviruses are capable of infecting distantly related eukaryotic cells. For example, *Orthotospovirus* (the tomato spotted wilt virus (*Bunyavirales*, *Tospoviridae*)) can replicate in both plant and insect cells [10,11].

The kinetoplastid flagellates of the family Trypanosomatidae are a eukaryotic group, whose viruses recently started attracting attention [12]. Trypanosomatids are obligate parasites of invertebrates, vertebrates, and plants [13]. They either have one or two hosts in their life cycle (monoxenous and dixenous species, respectively) [14,15,16]. Dixenous trypanosomatids originate from their monoxenous relatives and many of them are of medical or economic importance [17,18,19].

Members of the genus *Leishmania* infect vertebrates; they are transmitted by phlebotomine sand flies or, possibly, biting midges and cause a variety of diseases collectively named leishmaniases [20]. These diseases manifest with a wide spectrum of clinical symptoms from relatively harmless skin lesions to fatal cases involving failure of visceral organs. Currently, the genus *Leishmania* is subdivided into four subgenera: *Leishmania* (*Leishmania*), *L.* (*Mundinia*), *L.* (*Sauroleishmania*), and *L.* (*Viannia*) [21,22]. These groups are phylogenetically distinct and differ in host specificity or clinical symptoms. The recently established subgenus *Mundinia* is the most understudied one [23,24].

Thus far, only the representatives of the subgenera *Viannia* and *Leishmania* were extensively screened for viral presence, resulting in the discovery of *Leishmania* RNA viruses (LRVs). The first virus of this group was documented in *L.* (*V.*) *guyanensis* more than 30 years ago [25]. This double-stranded RNA (dsRNA) virus is classified as *Leishmaniavirus* within the family *Totiviridae* based on sequence similarity to the yeast L-A totivirus [26]. The genus *Leishmaniavirus* is subdivided into LRV1, infecting New World *Leishmania* (*Viannia*) [27,28], and LRV2 described from Old World *Leishmania* (*Leishmania*) [29,30,31]. Recently, new representatives of this viral genus were unexpectedly found in unrelated trypanosomatids, members of the monoxenous genus *Blechomonas* parasitizing fleas [32].

An increased interest in leishmaniaviruses was stimulated by the discovery that LRV1 presence may augment pathogenicity of some New World *Leishmania* species. It was shown that viral dsRNA interacts with Toll-like receptor 3 (TLR3) in the parasitophorous vacuole of a macrophage, initiating production of pro-inflammatory cytokines, including interferon-β [33] and subverts innate immunity via TLR3-mediated NLRP3 (NACHT, LRR and PYD domains-containing protein 3) inhibition of inflammasomes. This, in turn, leads to chronic inflammation that counteracts anti-leishmanial immune response and contributes to the metastatic potential of *Leishmania* [34,35]. It is argued that in this way the virus confers a selective advantage to *Leishmania*, resulting in its retention [36,37]. Only two strains of *L.* (*Mundinia*) *enriettii* were tested for LRV presence by PCR and both documented as negative [23].

No viruses other than LRVs were found in *Leishmania* spp [12]. At the same time, recent studies reveal numerous bunyaviruses infecting other trypanosomatids, including monoxenous relatives of *Leishmania* [38,39]. They all have a typical tripartite genome arrangement, although their M segment is markedly reduced in size and amino acid sequences of the M-encoded putative glycoprotein are extremely divergent. Sequences of their RDRPs and terminal complementary repeats are closest to those of *Phenuiviridae*. Leishbunyaviruses (LBVs, proposed family Leishbunyaviridae) form a single and well separated clade on a *Bunyavirales* tree, suggesting that they acquired the ability to infect trypanosomatids only once. Comparison of LBV and trypanosomatid phylogenies revealed cases of both co-evolution and horizontal viral transmissions [32,38].

In this work, we describe the first *Leishmania*-infecting leishbunyavirus as the first non-LRV virus in trypanosomatids of this genus.

## 2. Materials and Methods

### 2.1. Parasite Culture, DNA Isolation, and Verification of Species Identity

The following *Leishmania* (*Mundinia*) strains were used in this study: *L.* (*M.*) *enriettii* MCAV/BR/45/LV90, *L.* (*M.*) *macropodum* MMAC/AU/2004/AM-2004, *L.* (*M*.) *orientalis* MHOM/TH/2007/PCM2, and *L.* (*M.*) *martiniquensis* MHOM/MQ/92/MAR1. Promastigotes were cultured in modified M199 media supplemented with 1 mg/mL biotin, 0.5 mg/mL biopterin (both from Sigma-Aldrich, St. Louis, MO, USA), 2.5 µg/mL of hemin (Jena Bioscience GmbH, Jena, Germany), 1× MEM vitamin solution, 10% heat-inactivated fetal bovine serum, 500 units/mL of penicillin, and 0.5 µg/mL of streptomycin (all from Thermo Fisher Scientific, Waltham, MA, USA).

Total genomic DNA was isolated from 10 mL of log-phase trypanosomatid cultures with the DNeasy Blood & Tissue Kit (Qiagen, Hilden, Germany) according to the manufacturer’s instructions. Small subunit rRNA gene was amplified using primers S762 and S763 [40], following the previously described protocol [41]. The obtained PCR fragments were sequenced directly at Macrogen Europe (Amsterdam, The Netherlands) using the primers 883F, 907R, S757, and A757 [42]. The identity of the strains was confirmed by BLAST analysis [43].

### 2.2. DsRNA Isolation and Next-Generation Sequencing

Total RNA was extracted from 10^8^ cells using TRIzol (Thermo Fisher Scientific), following the manufacturer’s guidelines. The dsRNA fraction was isolated from 200 µg of total RNA using the previously described DNase-S1 nuclease method [38] and visualized in 0.8% agarose gels. The abundance of fragments was analyzed using GeneTools v. 4.3.9 (Syngene, Cambridge, UK). RiboMinus libraries, prepared from the dsRNA sample, were sequenced on the Illumina HiSeq 2500 platform (Illumina, San Diego, CA, USA) at Macrogen Inc. (Seoul, South Korea).

### 2.3. Viral Sequence Assembly

Transcriptome assembly was carried out essentially as described earlier [32]. In brief, reads were trimmed with Trimmomatic v. 0.36 [44] and assembled de novo using Trinity v. 2.4.0 [45]. Minimal k-mer was set to 5, and other parameters were not changed. Read mapping was performed in Bowtie2 v. 2.3.4.1 [46] and SAMtools v. 1.8 [47], and the coverage was calculated using BEDTools v. 2.25 software [48]. Viral segments were identified by BLAST searches of the 100 most abundant transcripts. Borders of viral segments were determined based on coverage value (with 10 reads per base as the threshold) and presence of specific terminal sequences. To obtain the terminal complementary sequences, original reads were trimmed with BBduk and mapped with BBmap (https://jgi.doe.gov/data-and-tools/bbtools/) to viral contigs assembled previously. GenBank accession numbers for the L, M, and S segment sequences are MK356554, MK356555, and MK356556, respectively.

### 2.4. Prediction of Functional Elements

The search for ORFs in the viral contigs was performed using NCBI ORFfinder [49] with the minimal ORF length set to 150 nt. The identification of the RDRP domain was done using the NCBI Conserved Domain Search [50]. Predictions of the transmembrane domains and membrane-targeting signal peptides were made using the TMHMM v. 2.0 (www.cbs.dtu.dk/services/TMHMM/), TMPred [51], Phobius [52], MEMSTAT3 on PSIPRED server [53], and SignalP v. 4.1 [54] software packages. *N*-glycosylation sites were identified with NetNGlyc 1.0 Server (www.cbs.dtu.dk/services/NetNGlyc/).

### 2.5. Phylogenetic Analyses

Full-length amino acid sequences of *Leishbunyaviridae* and *Phenuiviridae* RDRPs were aligned using MAFFT v. 7.313 E-INS-i algorithm [55]. The alignment was trimmed in TrimAl v. 1.4 with “automated1” algorithm [56], producing a matrix with 1772 amino acid positions that was used for phylogenetic reconstructions. Maximum likelihood analysis was performed in IQ-TREE v. 1.6.1 [57]. The best amino acid substitution model, LG with rate heterogeneity across sites approximated using proportion of invariant sites and 4 categories of discrete Γ distribution (+ I + G4), as well as the empirical amino acid frequencies (+ F), was selected by both corrected Akaike information criterion and Bayesian information criterion in the built-in ModelFinder [58]. Statistical supports for the branches were generated by running 1000 thorough bootstrap replicates. Bayesian inference was accomplished in MrBayes v. 3.2.6 [59] with the same substitution model and estimated during the run using “mixed” prior (resulting in 1.0 posterior probability of LG) and other model parameters specified above. The analysis was run for 1,000,000 Monte-Carlo Markov chain generations with default settings. For the comparison of nucleoproteins and glycoproteins, the respective alignments were prepared and trimmed in the same way as described above resulting in 163 and 190 aa data matrices. Maximum likelihood analysis for the nucleoproteins was performed similarly to the RDRPs. For the glycoproteins, pairwise p-distances were estimated in MEGA X [60].

### 2.6. Negative-Stain Transmission Electron Microscopy

In brief, gradient-purified virus samples were applied to a carbon-coated copper grid, stained with molybdenum acetate, and examined under a Philips 201C transmission electron microscope as described previously [38].

### 2.7. Treatment with Ribavirin

Virus-positive *L.* (*M.*) *martiniquensis* culture was treated with 2 mM of ribavirin (Sigma-Aldrich) for 4 weeks. The cultures were passaged weekly and the viral loads were measured by RT-qPCR in the LightCycler480 (Roche Life Science, Penzberg, Germany) as described previously [61,62] using the SYBR Green Master mix (Roche Life Science) and the following primer pairs: LBV_RDRP_for 5’-ggatcagcaaacaggagtcag-3’, LBV_RDRP_rev 5’-acatccaaaggctggcataca-3’; and 18S_for 5’–ttatggagctgtgcgacaag-3’, 18S_rev 5’-agtacgttctcccccgaact-3’. The cDNA was synthesized with random hexamer primers using the Super Script III-First strand synthesis kit (Thermo Fisher Scientific) following the manufacturer’s instructions. Then, 18S rRNA expression was used for normalization. The anti-viral treatment was stopped after 4 weeks, but the viral load was followed for 2 more weeks to ensure stable depletion.

### 2.8. Macrophage Infection

Mouse bone-marrow derived macrophages were infected as described previously [63] with modifications [64]. In brief, differentiated macrophages were cultured in complete RPMI-1640 medium supplemented with 10% fetal bovine serum (FBS), 50 units/mL of penicillin, 50 μg/mL of streptomycin, 2 mM of L-glutamine, and 0.05 mM of 2-mercapto-ethanol (all from Sigma-Aldrich) at 37 °C with 5% CO_2_. These cells were plated into CellStar 24-wells (Greiner Bio-One GmbH, Kremsmünster, Austria) at 4 × 10^5^ cells/mL. The stationary-phase *Leishmania* cells were added at a parasite to macrophage ratio of 6 promastigotes to 1 macrophage. After 2 h, cells were left either in complete RPMI-1640 or in the media combined with 50 U/mL IFN-γ (Bio-Rad) and 0.5 µg/mL LPS (Sigma-Aldrich) (classically stimulated macrophages) or with 25 ng/mL IL-4 (eBioscience/Thermo Fisher Scientific) (alternatively stimulated macrophages). Then, 72 h post infection, macrophages were lysed and amastigotes were counted by a hemocytometer after resuspension in the complete RPMI medium. All experiments were performed in two independent biological replicates and samples were analyzed in triplicate. Statistical analysis was done with a generalized linear model of the negative binominal distribution.

Ethics statement: Animals were maintained and handled in the animal facility of Charles University in Prague in accordance with institutional guidelines and Czech legislation (Act No. 246/1992 and 359/2012 coll. on protection of animals against cruelty in present statutes at large), which complies with all relevant European Union guidelines. All the experiments were approved by the Committee on the Ethics of Laboratory Experiments of the Charles University and were performed under permission No. MSMT-31114/2015-13 of the Czech Ministry of the Environment. All efforts were made to minimize the number and suffering of experimental animals during the study.

## 3. Results

### 3.1. Viral dsRNA in Leishmania (M.) martiniquensis

**Figure 1:**
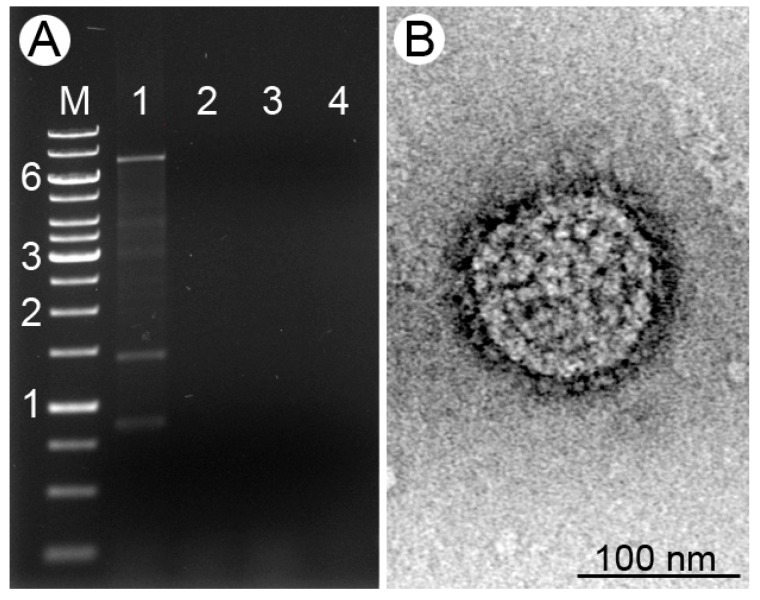
(**A**) Screening of double-stranded RNAs (dsRNAs) in *Leishmania* (*Mundinia*) spp. M, GeneRuler 1-kb DNA ladder. Indicated sizes are in kilobases: 1, *L.* (*M.*) *martiniquensis* MHOM/MQ/92/MAR1; 2, *L.* (*M.*) *enriettii* MCAV/BR/45/LV90; 3, *L.* (*M.*) *macropodum* MMAC/AU/2004/AM-2004; 4, *L.* (*M*.) *orientalis* MHOM/TH/2007/PCM2. (**B**) Negative-stain transmission electron micrographs of the virus particle isolated from *L.* (*M.*) *martiniquensis*. Scale bar is 100 nm.

**Figure 2:**
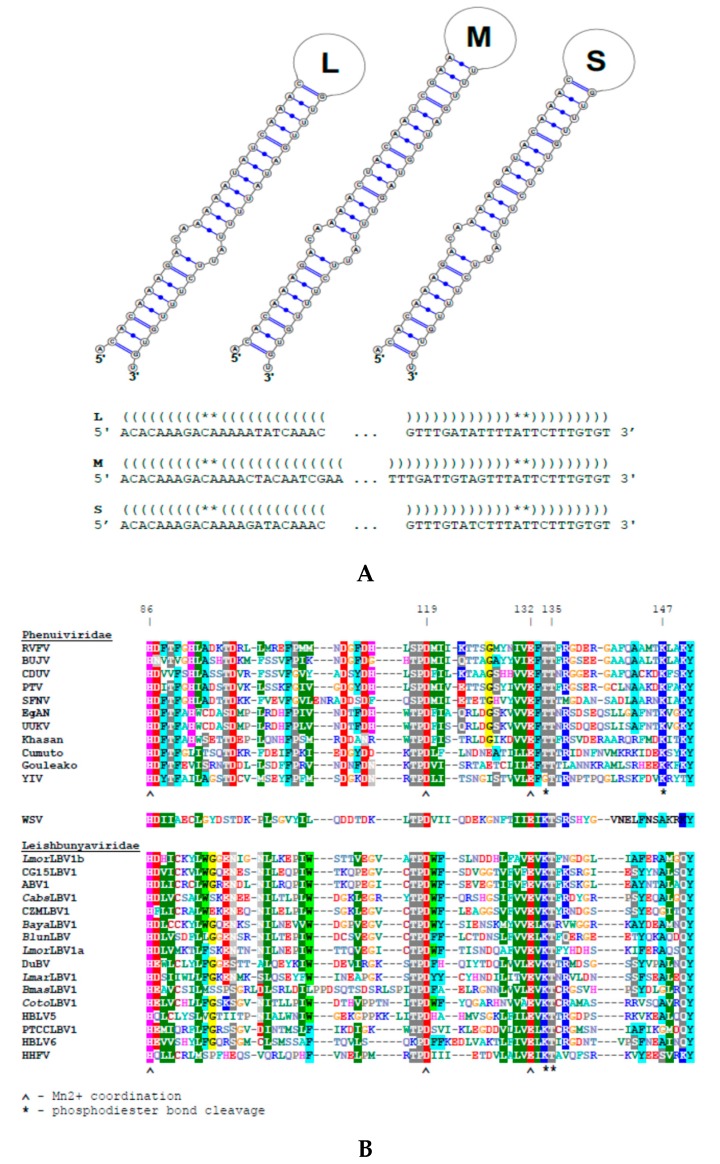
Structural features of *Lmar*LBV1. (**A**) Secondary structures and complementary sequences on 5′ and 3′ ends of the *Lmar*LBV1 L, M, and S RNA segments predicted by IPknot. (and) depicting complementary nucleotides forming the stem, *-non-complementary nucleotides forming a bulge. (**B**) Amino acid alignment of the N-terminal endonuclease domain of RDRP of *Leishbunyaviridae* and *Phenuiviridae*. Functionally important residues are marked with arrowheads. Numbering of positions in alignment are indicated as in *Lmar*LBV1 polymerase protein. Shading: ≥80% identity within Phenui and Leishbunyaviruses + Wuhan Spider virus (LBV+WSV).

Four isolates of four different species of the leishmanial subgenus *Mundinia* were screened for the presence of dsRNA viruses. In one of these isolates, *L.* (*M*.) *martiniquensis* MHOM/MQ/92/MAR1, we documented the presence of three major dsRNA bands designated as L, M, and S for large, medium, and short, respectively (Figure 1A). This sample was sequenced using the Illumina HiSeq platform, yielding 5.4 Gbp of sequence data. The three viral contigs (6.1, 1.2, and 0.7 kb long) were highly abundant (60.1 to 354.2 fold above the average RPKM (Reads Per Kilobase per Million mapped reads) value), which facilitated their quick and reliable identification. Each contained a single ORF; 2012, 334, and 165 aa long in the L, M, and S fragments, respectively. As previously reported for other leishbunyaviruses [32], the proportions of particular viral segments were not even. As compared to the L RNA, the S segment was about six-fold more abundant (Table 1 and Table S1). This is in agreement with the higher demand for the S RNA-encoded nucleoprotein in vRNP formation. The sequences of all three viral segments were complete and included both 5′ and 3′ terminal "panhandle" inverted repeats (5′-acacaaaga tctttgtgt-3′, Figure 2) necessary for replication, transcription, and translation in bunyaviruses [3]. The sequences of the identified terminal repeats were identical to those of other known LBVs [32,38].

BLASTp searches demonstrate that the ORF sequences within the L and S segments are very similar to the RDRPs (up to 43% identity with 96% coverage) and nucleocapsid proteins (up to 51% identity with 96% coverage) of leishbunyaviruses. The Conserved Domain search identified a bunyaviral RDRP domain (pfam04196) between aa 588 and 1306 in the L segment ORF with an E-value = 2.24e^−22^. The region between the aa 86 and 151 of the same ORF displayed organization typical for the endonuclease domain of leishbunyaviruses (Figure 2).

Consistent with the previously published data on LBVs [38], the search for the homologs encoded in the viral M segment did not return any hits with BLASTp, Conserved Domain search, PHYRE2, and HHpred software. The analysis of the M segment-encoded glycoprotein with TMHMM, TMPred, and Phobius did not identify any transmembrane domains (TMDs) in the viruses under study. However, like in other LBVs and consistent with the glycoprotein annotation, SignalP detected the N-terminal membrane insertion peptide (with cleavage site between aa 21 and 22) and NetNGlyc predicted two *N*-glycosylation sites (at amino acid positions 34 and 237) in M segment sequences. Previously, similar results were obtained for *Lepmor*LBV1, whereas in *Cabs*LBV1, *Coto*LBV1, and the LBVs of *Blechomonas* spp., two TMDs were predicted in this segment [32,38]. We posit that such discrepancy could be explained by extreme sequence divergence preventing unambiguous identification of these elements. Application of a more sensitive algorithm, MEMSAT3, predicted one TMD in the virus investigated here, *Lepmor*LBV1, as well as in Duke bunyavirus, which was not analyzed before. Similar to typical bunyaviruses, other LBVs have three TMDs in their glycoprotein ORFs. Analyses presented above suggest that the virus under investigation, as other bunyaviruses, can utilize host machinery for glycoprotein synthesis and virion assembly [65,66]. Indeed, the negatively stained transmission electron microscopy on purified virions from *L.* (*M*.) *martiniquensis* demonstrate the typical envelope with evenly spaced surface projections (Figure 1B).

In summary, we demonstrate that the new virus possesses many characteristic features of leishbunyaviruses and, therefore, we named it *Leishmania martiniquensis* leishbunyavirus 1 (*Lmar*LBV1).

### 3.2. Phylogeny

**Figure 3:**
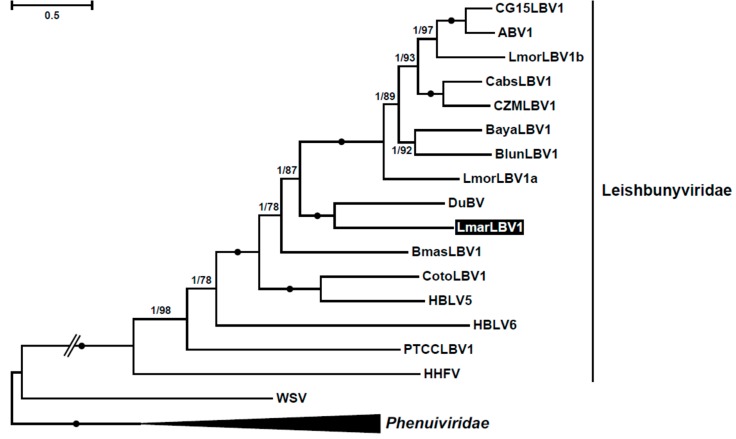
RDRP-based maximum likelihood reconstruction of leishbunyaviruses’ phylogeny. Double-crossed branch is at 50% of its original lengths. Branch supports are Bayesian posterior probability and maximum likelihood bootstrap, respectively. Black circles indicate maximal (1/100) statistical supports. The scale bar indicates the number of substitutions per site. The tree was rooted with the sequences of *Phenuiviridae*. *Lesihmania martiniquensis* leishbunyavirus 1 (*Lmar*LBV1) described here is highlighted in black. Abbreviations and GenBank accession numbers are in Table S2 [32].

The amino acid sequence of the RDRP was used in the phylogenetic inference of *L. martiniquensis* leishbunyavirus, using sequences of related *Phenuiviridae* as an outgroup (Figure 3). *Lmar*LBV1 was nested within the clade Leishbunyaviridae with its closest relative being the Duke bunyavirus [67], which presumably infects a trypanosomatid from bees [38]. These two species proved to be sister to a big cluster of viruses from various monoxenous trypanosomatids. Judging by its phylogenetic position, we propose that *L. martiniquensis* acquired leishbunyavirus from a monoxenous trypanosomatid.

Although the glycoprotein sequences of LBVs are quite divergent, we perceived that the C-terminal part in some of them displayed conserved residues (Figure S1). Of note, all these viruses were those with one predicted TMD. Moreover, the sequence of this region in *Lmar*LBV1 is more similar to that in *Lepmor*LBV1s, than in DuBV, its closest relative according to the RDRP tree (Figure 3). Indels, rather than amino acid substitutions, distinguished these glycoprotein sequence fragments. *Lmar*LBV1 had 28 and 77 indels compared to *Lepmor*LBV1s and DuBV, respectively. The analysis of *p*-distances in trimmed alignments of the full glycoprotein sequences of all available species also showed markedly higher similarity between *Lmar*LBV1 and *Lepmor*LBV1s than between *Lmar*LBV1 and DuBV (Table S3). Of interest, although nucleoprotein sequences were too short for reliable phylogenetic analysis, they grouped *Lmar*LBV1 with DuBV, similarly to the RDRP-based tree (Figure S2).

### 3.3. LmarLBV1 Has Minor Effect on *Leishmania* Infectivity In Vitro

**Figure 4:**
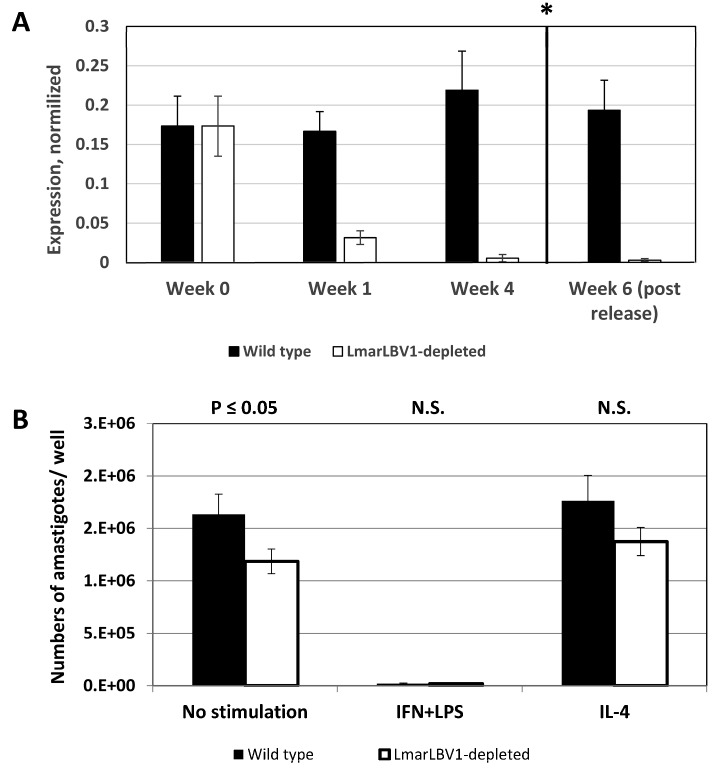
*Lmar*LBV1 facilitates *Leishmania* infection in vitro. (**A**) Establishment of isogenic, virus-depleted line of *L.* (*M.*) *martiniquensis* MHOM/MQ/92/MAR1. The treatment with ribavirin was stopped after four weeks (asterisk), but the viral load remained low in *Lmar*LBV1-depleted parasites. (**B**) Macrophage infection in vitro. The average number of parasite per well was calculated for the wild type and in *Lmar*LBV1-depleted *L.* (*M.*) *martiniquensis* infecting non-stimulated, classically (LPS/IFN-γ) or alternatively (IL-4) stimulated primary murine macrophages. Data are summarized from two independent biological replicates (three technical replicates each). The error bars indicate standard deviations. N.S. = not statistically significant. *p* ≤ 0.05.

To assess the role of *Lmar*LBV1 in *Leishmania* biology, we first established an isogenic line of *L.* (*M*.) *martiniquensis* MHOM/MQ/92/MAR1 depleted of leishbunyavirus using ribavirin (Figure 4A). After four weeks of treatment, the viral load (as judged by RT-qPCR) was significantly diminished in the treated, compared to the untreated cells. Importantly, it stayed low even after the treatment was stopped (Figure 4A, asterisk), indicating that depletion was not transient.

Wild type and *Lmar*LBV1-depleted *L.* (*M.*) *martiniquensis* were used to infect non-stimulated, classically (LPS/IFN-γ), or alternatively (IL4) stimulated primary murine macrophages to assess early stages of infection. As expected, the infection level in the classically stimulated macrophages was significantly lower compared to either non-stimulated or IL-4-treated cells. Importantly, parasites, which were depleted of virus, were less infective, compared to their wild type kin (Figure 4B). The effect of viral presence is minor, yet it is statistically significant and may point out the potential role of *Lmar*LBV1 in *L.* (*M.*) *martiniquensis* biology.

## 4. Discussion

**Figure 5:**
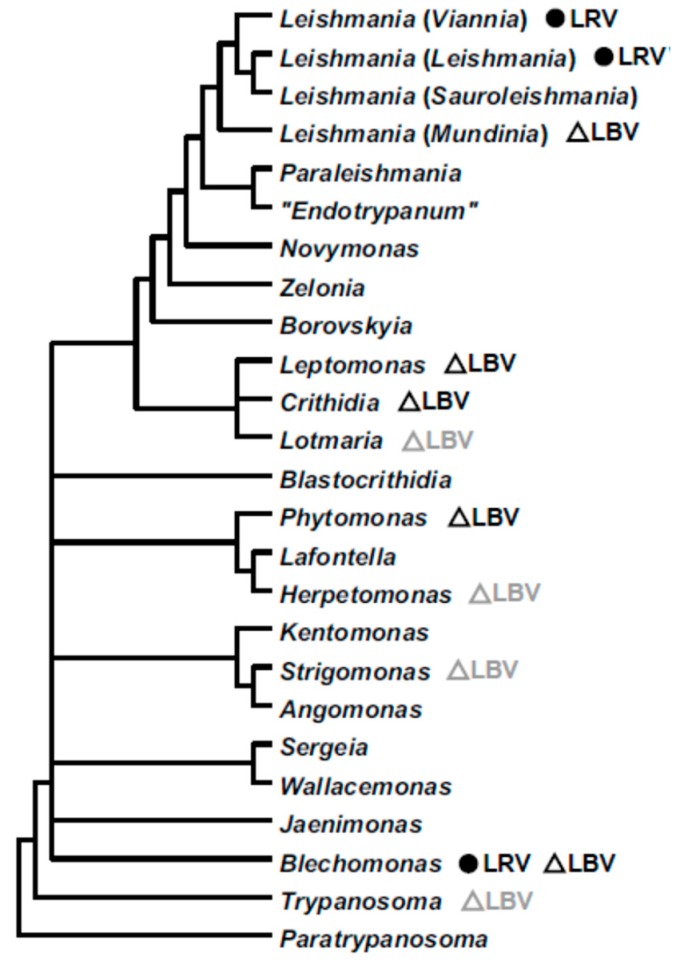
A schematic phylogenetic tree of the family Trypanosomatidae (modified from [13]), demonstrating the distribution of leishbunyaviruses (triangles) and leishmaniaviruses (circles) over the genera of these flagellates. Viruses identified in metatranscriptomes of trypanosomatid-infected insects [38] are shown in grey.

In this study, we describe the first leishbunyavirus of *Leishmania*. Previously, these viruses were discovered in monoxenous trypanosomatids of the subfamily Leishmaniinae (mainly in *Crithidia* spp.), genus *Blechomonas* (subfamily Blechomonadinae), as well as in one plant-infecting dixenous *Phytomonas* sp. (Figure 5) [32,38,39]. Leishbunyaviral sequences are also found in metatranscriptomes of insects infected by flagellates of other trypanosomatid genera, such as *Strigomonas*, *Herpetomonas,* and *Trypanosoma* (Figure 5, light grey) [38]. This is the most widespread and species-rich group of RNA viruses in trypanosomatids known to date. This fact, along with the discordance of viral and trypanosomatid phylogenies documented in the previous studies [32,38], strongly suggests that host-to-host transition is significantly facilitated in this group of viruses. It is explained when taking into account two facts: (i) LBVs are able to form membrane-bound viral particles [68] and (ii) the flagellar pocket of trypanosomatids is an organelle-governing intensive exchange with the milieu by endo- and exocytosis [69,70,71,72]. The documented particles of LBVs measure about 100 nm [38] corresponding to the typical size of clathrin-coated endocytic vesicles in trypanosomatids [73]. Interestingly, clathrin-mediated endocytosis is the general route for an uptake of bunyaviruses [74]. Bunyaviruses evolved to utilize the eukaryotic endomembrane system for virus assembly and spreading. Apparently, LBVs use the same strategy in trypanosomatids.

Infectivity and formation of viral particles in bunyaviruses depend on glycoproteins, type I transmembrane proteins that are proteolytically processed and glycosylated in the ER [3,8]. Their C-terminal cytoplasmic domains are thought to bind viral ribonucleoproteins and play a crucial role in genome packaging [75,76,77], whereas the N-terminal ectodomains are involved in receptor recognition and membrane fusion [65,78,79]. In leishbunyaviruses, the M segments and putative glycoproteins encoded within them are significantly reduced in size, extremely divergent, and sometimes contain a reduced number of transmembrane domains. We hypothesize that such a layout reflects a reduced functionality of these proteins and potential broad specificity of viral infection, which explains their facilitated host-to-host transition. It was demonstrated that extended deletions in the bunyaviral glycoprotein N-terminus (ectodomain) do not prevent cell fusion and transport to the Golgi, but lead to attenuation of viruses [80]. This illustrates the propensity of these proteins to undergo reduction. The opportunity to be inherited vertically probably removes the need for efficient proliferation and infection and may even make such properties undesirable.

Transfer of viruses between different species of trypanosomatids is possible because of coinfections, which are quite common in these parasites [81,82,83,84,85]. Coinfections were previously reported for *Leptomonas moramango* [39]. Here we did not observe viral coinfection but revealed a putative consequence of such an event—re-assortment of genomic segments. This assumption arises from discordance of phylogenies of proteins from the L and S segments on one hand, and the M segment on the other. The RDRP and nucleocapsid of *Lmar*LBV1 are closely related to their counterparts in DukeBV, whereas its glycoprotein is more similar to the corresponding proteins of *Lepmor*LBV1a and *Lepmor*LBV1b.

*Lmar*LBV1 is the first non-LRV virus discovered in *Leishmania.* It was found in one of the members of the most enigmatic subgenus of these flagellates—*Mundinia.* Although the first species was characterized over 70 years ago, the subgenus itself was established only recently [21]. For the moment, this taxon contains four described species: *L.* (*M.*) *enriettii*, *L.* (*M*.) *macropodum*, *L.* (*M*.) *martiniquensis*, and *L.* (*M*.) *orientalis* [86,87,88,89,90,91]. The first two infect guinea pigs and kangaroos, respectively, while the remaining two are isolated from humans. In contrast to other human-infecting *Leishmania*, which use sand flies as their vectors, these flagellates may be transmitted by biting midges [92,93]. Host switching may have shaped the genome evolution in these flagellates [94]. While the parasitofauna of biting midges is understudied, several species of monoxenous trypanosomatids are documented in these insects [95,96,97]. This is in agreement with our proposal that *Lmar*LBV1 originates from LBVs of monoxenous trypanosomatids.

*Leishmania martiniquensis* is frequently found in skin lesions of immunocompromised patients indicating that it may be an opportunistic pathogen [98,99,100,101]. However, recent analysis of multiple records in Thailand and Myanmar reveals that neither the presence nor the severity of the infection is necessarily associated with HIV [87]. Notably, the clinical manifestations range from asymptomatic infection and various types of lesions to visceral disease. Previously, it was demonstrated that LRV1 boosts virulence of *Leishmania guyanensis* in humans [33,34,102,103]. The discovery of a virus in *L. martiniquensis* poses an important question on whether it also influences the pathogenicity of this parasite. We demonstrate that the presence of *Lmar*LBV1 is slightly beneficial for *Leishmania*. The molecular mechanism of such facilitation may be non-specific, since it was recently shown that simultaneous inoculation of virus-negative *L. guyanensis* and Toscana virus (*Bunyavirales*, *Phenuiviridae*) increases footpad swelling and parasite burden in mice, reminiscent of the reaction to the LRV1-positive *L. guyanensis* [35]. Although it was not shown experimentally, the presence of the membrane-bound viral particles in LBVs suggest that they can be shed by trypanosomatid cells. This way, *Lmar*LBV1 can interact with the immune system of a vertebrate host, increasing the severity of leishmanial infection. Our results signify the need for a systematic exploration of trypanosomatid viromes.

## Figures and Tables

**Table 1:** Molecular data for the identified RNA sequences^1^.

Viral Sequences	Accession	Length, bp	ORF, AA	RPKM
*Lmar*LBV1 S	MK356556	721	165	6,600.13
*Lmar*LBV1 M	MK356555	1,244	334	1,368.42
*Lmar*LBV1 L	MK356554	6102	2,012	1,131.22

^1^ See also Table S1. ORF: Open Reading Frame, AA: Amino Acids, RPKM: Reads per kilobase per million mapped reads.

## Supplementary Materials

The following are available online at https://www.mdpi.com/1999-4915/12/2/168/s1, Figure S1: Alignment of C-terminal part of the putative glycoproteins, possessing only one predicted transmembrane domain. Columns with ≥3 functionally similar amino acids are shaded, those with four functionally similar amino acids are marked with asterisk. Figure S2: Maximum likelihood phylogenetic tree of leishbunyaviral nucleoproteins. Numbers at branches indicate bootstrap support, values below 50 are not shown. The tree is rooted in agreement with RDRP-based reconstruction. Table S1: Summary statistics for RNA-seq data. Table S2: RDRP sequences of *Bunyavirales* with working abbreviations of viral names used in phylogenetic inferences. Table S3: Pairwise p-distances between glycoprotein sequences in *Leishbunyaviridae*.
