# Supplementary material for: The First Non-LRV RNA Virus in Leishmania
Source: Viruses. 2020 Feb 2;12(2):168. doi: 10.3390/v12020168 (PMC7077295; doi:10.3390/v12020168)
Supplement: Supplementary file 1 [file viruses-12-00168-s001.pdf]

Alignment of C-terminal part of the putative glycoproteins

```
                                          *     *           *  *           * *
LmarLBV1_gp   MWYPSRATDHYNLYIDKDTFIVSSKISYGHTVTLLTCRNFKDENITLSGRVVYLRTNIYQRCILAGERLE
LmorLBV1b_gp  VFYPERITENYDFDVDDFAVTIWTKGGSVSSAIRARCKDVYGTDYLVTGNSVRITTNDTLVCLITGTTFI
LmorLBV1a_gp  VVYPSEILQNYKIDVDPDGVVISTSKGDWSTAIRAECANYKGLRRHLIGTTLRIRSKDSLACLITGSRQT
DuBV_gp       ---------RGYNNDF------------WATGYRGYCANSRD------GLTISFVGKRSFKMSVTGSVIS

                                                        *          *
LmarLBV1_gp   Y----------------------FDDFVAHHRRL------SQSG--------IVLNDGYQGQKQANFR-Y
LmorLBV1b_gp  W----------------------KPNHETPLYFR------MDSKCMDHGRVSLMLD-------------N
LmorLBV1a_gp  L----------------------YKDITMRYAFE------SKTGCETKLKARQLFD-------------S
DuBV_gp       FCFDDKGKMYGSPVWGPYNWEHEWNDMTCYVYRAGVEMVPAATNCVAEKECLRLLNPTYQ-----VVRFH

                *    *
LmarLBV1_gp   ISTPTIILALGTGERDQVR-------AMQDCLENRNEGTWLYSFQ------
LmorLBV1b_gp  WHTPSFILSVGPYKRRRI--------LEELCRENRNGGFFRNGYGQQCYVT
LmorLBV1a_gp  WYSPSFFLSVGPVDRLPV--------LYESCKDNQDGEFFRDDYKQQCYAS
DuBV_gp       WSSSRFDLDKVVCDELDKRVNIASAEMTEAAKNIGNQGAAGNDICHRALSN
```

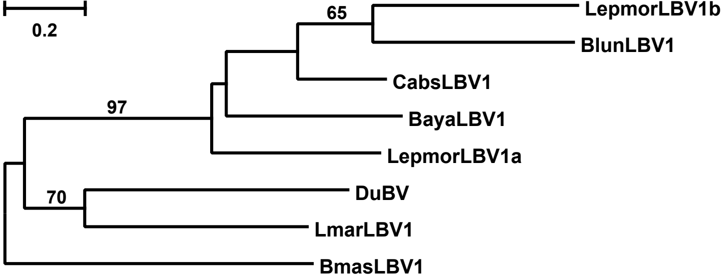
0.2
65
LepmorLBV1b
BlunLBV1
CabsLBV1
97
BayaLBV1
LepmorLBV1a
70
DuBV
LmarLBV1
BmasLBV1

Viral sequences	length (bp)	ORF, AA	RPKM	RPM (proportional to the brightness on EtBr gel)	x-fold above average RPKM	mass units (gel-estimated)	gel-estimated molar equivalents	Fold increase relative to L (gel data)	Fold increase relative to L (RNA-seq data)	Fold discrepancy between gel and RNA-seq for S and M
LmarLBV segment S	721	165	6600.13	4758.69373	267.536684	7.641	10.59778086	6.132542324	5.834523788	1.051078468
LmarLBV segment M	1244	334	1368.42	1702.31448	55.4689907	5.942	4.776527331	2.76399903	1.209685119	2.284891322
LmarLBV segment L	6102	2012	1131.22	6902.70444	45.8540738	10.545	1.728121927	1	1	1

average RPKM in assembly = 24.67

viral contigs are in top ~15 most covered (excluding isoforms)

Other sequences	contig name	length (bp)	RPKM	RPM (should be proportional to the brightness on EtBr gel [unless there is uneven coverage])	
28S ribosomal RNA gene, complete sequence Leishmania panamensis strain UA946 JQ648649.1	TRINITY_DN2698_c14_g10_i1_len_874	874	72325.2	63212.2	
28S ribosomal RNA (LSU-alpha) (LDBPK_27rRNA4), partial rRNA Leishmania donovani XR_002966728.1	TRINITY_DN1879_c0_g1_i1_len_246	246	50433.3	12406.6	
18S ribosomal RNA gene, complete sequence, Leishmania sp. MHOM/MQ/92/MAR1 AF303938.1	TRINITY_DN2699_c5_g10_i1_len_1552	1552	50044.9	77669.7	
28S ribosomal RNA (LSU-alpha) (LDBPK_27rRNA4), partial rRNA Leishmania donovani XR_002966728.1	TRINITY_DN2700_c7_g3_i1_len_1490	1490	12360.5	18417.1	
No BLASTn hits	TRINITY_DN2562_c6_g1_i1_len_207	207	3929.16	813.336	
Leishmania mexicana MHOM/GT/2001/U1103 complete genome, chromosome 5 FR799558.1	TRINITY_DN2621_c4_g1_i13_len_842	842	2991.21	2518.6	uneven coverage
putative kinesin partial mRNA Leishmania infantum JPCM5 XM_001464597.2	TRINITY_DN2537_c2_g2_i13_len_274	274	2057.68	563.804	
No BLASTn hits	TRINITY_DN2645_c5_g2_i2_len_251	251	1391.87	349.359	
putative proteophosphoglycan ppg3 partial mRNA Leishmania infantum JPCM5 XM_003392744.1	TRINITY_DN2457_c4_g1_i1_len_2355	2355	1291.21	3040.8	uneven coverage

partial hit (most of the seq has no DB homologs): TU1 small subunit ribosomal RNA gene, partial sequence Leptomonas pyrrhocoris isolate MK056191.1	TRINITY_DN2546_c0_g1_i3_len_671	671	1129.37	757.807	uneven coverage
dipeptidyl-peptidase 8-like serine peptidase + some intergenic region Leishmania mexicana MHOM/GT/2001/U1103 XM_003874544.1	TRINITY_DN2654_c2_g11_i1_len_4252	4252	1076.84	4578.72	uneven coverage
partial hit (most of the seq has no DB homologs): Leishmania braziliensis MHOM/BR/75/M2904 genome assembly, chromosome: 28	TRINITY_DN2658_c4_g10_i2_len_884	884	1007.49	890.621	uneven coverage
putative RNA binding protein partial mRNA Leishmania mexicana MHOM/GT/2001/U1103 XM_003877869.1	TRINITY_DN2459_c1_g4_i5_len_1591	1591	797.698	1269.14	

Clade	Short name	Virus name	RDRP Accession
Phlebovirus	CDUV	Chandiru virus	AEA30057.1
Phlebovirus	PTV	Punta Toro phlebovirus	ALL45372.1
Phlebovirus	BUJV	Bujaru virus	API68880.1
Phlebovirus	SFNV	Sandfly fever Naples virus	CAA48478.1
Phlebovirus	RVFV	Rift Valley fever virus	ABD51507.1
Phlebovirus (uukuniemi grour	EgAN	EgAN 1825-61 virus	AEL29654.1
Phlebovirus (uukuniemi grour	UUKV	Uukuniemi phlebovirus	BAA01590.1
Phlebovirus (uukuniemi grour	Khasan	Khasan virus	AII79370.1
Goukovirus	Cumuto	Cumuto virus	AHH60917.1
Goukovirus	Gouleako	Gouleako virus	AEJ38175.1
Goukovirus	YIV	Yichang Insect virus	AJG39273.1
Leishbunyaviridae	ABV1	Apis bunyavirus 1	ARO50045.1
Leishbunyaviridae	CG15LBV1	Crithidia sp. G15 leishbunyavirus 1	ASN64747.1
Leishbunyaviridae	LmorLBV1b	Leptomonas moramango leishbunyavirus 1l	ANJ59513.1
Leishbunyaviridae	CZMLBV1	Crithidia sp. ZM leishbunyavirus 1	ASN64749.1
Leishbunyaviridae	CabsLBV1	Crithidia abscondita leishbunyavirus 1	AOA33725.1
Leishbunyaviridae	LmorLBV1a	Leptomonas moramango leishbunyavirus 1a	ANJ59510.1
Leishbunyaviridae	DuBV	Duke bunyavirus	ARE30258.1
Leishbunyaviridae	HBLV5	Hubei bunya-like virus 5	APG79301.1
Leishbunyaviridae	CotoLBV1	Crithidia otongatchiensis leishbunyavirus 1	ASN64748.1
Leishbunyaviridae	HBLV6	Hubei bunya-like virus 6	APG79326.1
Leishbunyaviridae	PTCCLBV1	Phytomonas sp. TCC231 leishbunyavirus 1	AUF41956.1
Leishbunyaviridae	HHFV	Huangshi Humpbacked Fly virus	AJG39239.1
Leishbunyaviridae	WSV	Wuhan Spider virus	AJG39269.1
Leishbunyaviridae	BayaLBV1	Blechmonas ayalai leishbunyavirus 1	AYD61653.1
Leishbunyaviridae	BlunLBV1	Blechmonas luni leishbunyavirus 1	AYD61649.1
Leishbunyaviridae	BmasLBV1	Blechomonas maslovi leishbunyavirus 1	AYD61658.1

LmarLBV1								
B09-1006_LBV	0.896							
B08-376_LBV	0.853	0.815						
CotoM	0.826	0.785	0.899					
B05-J13_LBV	0.844	0.825	0.892	0.833				
CabsLBV1	0.843	0.801	0.880	0.789	0.797			
LmorLBV1b	0.767	0.845	0.836	0.816	0.805	0.810		
LmorLBV1a	0.772	0.830	0.857	0.837	0.832	0.796	0.619	
DuBV	0.846	0.868	0.833	0.776	0.867	0.829	0.851	0.838
